# Cannabidiol Adverse Effects and Toxicity

**DOI:** 10.2174/1570159X17666190603171901

**Published:** 2019

**Authors:** Marilyn A. Huestis, Renata Solimini, Simona Pichini, Roberta Pacifici, Jeremy Carlier, Francesco Paolo Busardò

**Affiliations:** 1 Lambert Center for the Study of Medicinal Cannabis and Hemp, Institute of Emerging Health Professions, Thomas Jefferson University, Philadelphia, PA, USA;; 2 National Centre on Addiction and Doping, Istituto Superiore di Sanità, Rome, Italy;; 3 Unit of Forensic Toxicology (UoFT), Department of Anatomical, Histological, Forensic and Orthopedic Sciences, Sapienza University of Rome, Rome, Italy;; 4 Section of Legal Medicine, Università Politecnica delle Marche, Ancona, Italy

**Keywords:** Cannabidiol, adverse effects, toxicity, animal studies, *in vitro* studies, *in vivo* studies, studies in humans

## Abstract

***Background:*** Currently, there is a great interest in the potential medical use of cannabidiol (CBD), a non-intoxicating cannabinoid. Productive pharmacological research on CBD occurred in the 1970s and intensified recently with many discoveries about the endocannabinoid system. Multiple preclinical and clinical studies led to FDA-approval of Epidiolex^®^, a purified CBD medicine formulated for oral administration for the treatment of infantile refractory epileptic syndromes, by the US Food and Drug Administration in 2018. The World Health Organization considers rescheduling cannabis and cannabinoids. CBD use around the world is expanding for diseases that lack scientific evidence of the drug’s efficacy. Preclinical and clinical studies also report adverse effects (AEs) and toxicity following CBD intake.

***Methods:*** Relevant studies reporting CBD’s AEs or toxicity were identified from PubMed, Cochrane Central, and EMBASE through January 2019. Studies defining CBD’s beneficial effects were included to provide balance in estimating risk/benefit.

***Results:*** CBD is not risk-free. In animals, CBD AEs included developmental toxicity, embryo-fetal mortality, central nervous system inhibition and neurotoxicity, hepatocellular injuries, spermatogenesis reduction, organ weight alterations, male reproductive system alterations, and hypotension, although at doses higher than recommended for human pharmacotherapies. Human CBD studies for epilepsy and psychiatric disorders reported CBD-induced drug-drug interactions, hepatic abnormalities, diarrhea, fatigue, vomiting, and somnolence.

***Conclusion:*** CBD has proven therapeutic efficacy for serious conditions such as Dravet and Lennox-Gastaut syndromes and is likely to be recommended off label by physicians for other conditions. However, AEs and potential drug-drug interactions must be taken into consideration by clinicians prior to recommending off-label CBD.

## 1 INTRODUCTION

### 1.1 Cannabinoid Pharmacology

Δ9-tetrahydrocannabinol (THC) was shown to be the primary psychoactive compound in cannabis (marijuana) in 1964 by Gaoni and Mechoulam [1]. There were few advances in cannabinoid pharmacology until 1988, when Devane *et al*. identified the first CB1 cannabinoid receptor [2], quickly followed by the discovery of the CB2 peripheral receptor by Munro *et al*. in 1990 [3]. The CB1 and CB2 cannabinoid receptors were cloned by Matsuda *et al*. in 1992 [4] and Munro *et al*. in 1993 [3], respectively. However, the endogenous cannabinoid system may include additional cannabinoid G protein-coupled receptors (GPCR) GPR55, GPR18, and GPR119, transient receptor potential cation channels (TRP) TRPV, TRPA, TRPM, and TRPC and nuclear peroxisome proliferator-activated receptors (PPAR) [5]. Anandamide was the first identified endogenous cannabinoid ligand [6], but there are many other endocannabinoids including 2-arachidonylglycerol, N-palmitoyl ethanolamide, and N-oleoyl ethanolamide.

Cannabidiol (CBD or 2-[(6R)-6-isopropenyl-3-methyl-2-cyclohexen-1-yl]-5-pentyl-1,3-benzene-diol) was identified in an extract of Minnesota wild hemp by Adams *et al*. at the University of Illinois in 1940 [7], but its structure was not fully elucidated until 1963 [8]. To date, CBD’s mechanisms of action are not fully elucidated [9]. CBD modulates central nervous system (CNS) receptors such as CB1, CB2, serotonin 1A receptor (5-HT1A), TRPV1, and PPARγ, although it binds poorly to the THC-binding site on CB1 and CB2 cannabinoid receptors [10]. CBD may antagonize CB1 receptor function by negative allosteric modulation of the orthosteric receptor site [11-14]. CBD may be an inverse agonist at the CB2 receptor, partially explaining its anti-inflammatory properties [15], which also are supported by CBD PPARɤ activation [16]. High CBD doses activate TRPV1 receptors promoting anxiolytic effects [17]. CBD also increases serotoninergic and glutamatergic transmission through a positive allosteric modulation of 5-HT1A serotonin receptors [10]. 5-HT1A receptor activation is also involved in CBD neuroprotection in *in vitro* adult and rat newborn models of the acute hypoxic-ischemic brain [18].

CBD is metabolized in the liver and the intestine by cytochrome P450 (CYP) CYP2C19 and CYP3A4, and 5'-diphosphoglucuronosyltransferase (UGT) UGT1A7, UGT1A9, and UGT2B7 isoforms, mainly producing hydroxylated and carboxylated metabolites [19]. CBD inhibited barbiturate metabolism, increasing barbiturate-induced sleep duration in mice, and also phenazone hepatic metabolism [20] due to the inhibition of CYP3A and CYP2C microsomal enzymes [21]. Other research suggested that CBD also induced hepatic CYP3A, CYP2B, and CYP2C [22]. Later, CBD was shown to inhibit THC metabolic hydroxylation in humans. The pharmacokinetic interaction between THC and CBD may explain why CBD administration prior to THC potentiates THC effects [23].

The complexity of CBD pharmacology offers tremendous therapeutic potential but also the potential for AEs and drug-drug interactions.

### 1.2 Potential Therapeutic Effects of CBD

In 2017, the National Academies of Science, Engineering and Medicine evaluated all the published literature through August, 2016 on the potential therapeutic uses of cannabinoids [24]. They determined if there was conclusive evidence, substantial evidence, moderate evidence, limited evidence, or insufficient evidence for cannabinoids being an effective or ineffective therapy to treat chronic pain, cancer, chemotherapy-induced nausea/vomiting, appetite and weight loss, irritable bowel syndrome, epilepsy, spasticity of multiple sclerosis, Tourette syndrome, amyotrophic lateral sclerosis, Huntington’s disease, Parkinson’s disease, dystonia, Alzheimer’s disease/dementia, glaucoma, traumatic brain injury/spinal cord injury, addiction, anxiety, depression, sleep disorders, posttraumatic stress disorder, and schizophrenia. In addition, they reviewed the knowledge base using the same evidence categories for the health effects of cannabinoids and cancer, cardiometabolic risk, acute myocardial infarction, stroke, metabolic dysregulation, metabolic syndrome, diabetes, respiratory disease, immunity, injury and death, prenatal, perinatal, and postnatal exposure to cannabis, psychosocial, mental health, and problem cannabis use.

This review is not focused on therapeutic indications but rather on potential AEs, toxicities and drug-drug interactions that may accompany CBD therapeutics and that must be considered prior to off-label use of CBD for pathophysiology that has not yet been shown to respond effectively to CBD. However, to enable the reader to independently evaluate CBD’s AEs and toxicity, we briefly highlight some current research supporting CBD therapeutics.

### 1.3 Anti-epileptic

As early as 1980, the potential therapeutic effect of 200-300 mg/day CBD in patients with uncontrolled epilepsy was evaluated [25]. Patients tolerated CBD well, with no signs of toxicity or serious side effects detected. Seven of 8 subjects receiving CBD had fewer convulsive episodes, with 3 only partially improved. A 2018 meta-analysis concluded that CBD in conjunction with other anti-epileptic drugs decreased seizure frequency in patients with Dravet’s and Lennox-Gastaut syndromes or who experienced intractable seizures, although AEs occurred more frequently than placebo [26]. The US Food and Drug Administration (FDA) approved Epidiolex^®^ for the treatment of refractory epilepsy in 2018 [19, 27].

### 1.4 Anxiolytic

Multiple studies evaluated the potential therapeutic effect of CBD on anxiety, psychotic symptoms, and depression in humans since the 1980s, mostly showing mild AEs [28-35]. CBD effectively treated anxiety by activating limbic and paralimbic regions of the brain [30].

Interestingly, a single acute administration of a low 3 mg/kg CBD dose in mice had an anxiolytic effect, while repeated administration of a 3 or 10 mg/kg dose exerted antidepressant effects by cell proliferation and neurogenesis [36]. Conversely, CBD anxiolytic effects were not observed at higher 10 and 30 mg/kg CBD doses or after 15 days of 30 mg/kg/day dosing. The authors suggest that there is an inverted U-shaped dose-response curve for CBD’s effects on anxiety.

### 1.5 Antipsychotic Properties

CBD is extensively studied for its antipsychotic effects on schizophrenia [35, 37]. Leweke *et al*. noted that CBD moderately inhibits degradation of the endocannabinoid anandamide [38]. They performed a double-blind, randomized clinical trial of CBD *vs*. amisulpride, a potent antipsychotic, in acute schizophrenia. Both treatments were safe and significant clinical improvement was achieved, but CBD had a better side effect profile. CBD treatment significantly increased serum anandamide concentrations.

The safety and effectiveness of 1000 mg/day CBD in patients with schizophrenia were assessed [35]. These patients (n=43) with schizophrenia received 1000 mg/day CBD in addition to their existing antipsychotic medications. After 6 weeks of treatment, the CBD group had lower levels of positive psychotic symptoms (positive and negative syndrome scale (PANSS): treatment difference=21.4, 95% CI=22.5,20.2). CBD was well tolerated, and AEs were similar between the CBD and placebo groups.

Six-hundred mg oral CBD was evaluated for its effects on persecutory ideation and anxiety in a high paranoid trait group (n=32) 130 min before entering a virtual-reality scenario [39]. CBD had no impact on anxiety (Beck's anxiety inventory), or cortisol concentration, systolic blood pressure, and heart rate. In fact, in this study, a strong trend towards increased anxiety was documented and CBD had no effect on persecutory ideation.

### 1.6 CBD Neuroprotection

CBD’s anti-inflammatory and antioxidant properties may offer a new pharmacological approach for neuroprotection and a reduction in hippocampal volume loss [23, 40, 41]. CBD protects against hippocampal pathology following chronic frequent THC use [42]. This CBD restorative effect on hippocampal substructures suggests a therapeutic potential for other pathologies such as schizophrenia, Alzheimer’s disease, and major depressive disorder [40]. Indeed, in human studies for schizophrenia [35, 38] and Parkinson’s disease [43], and in animal studies for symptoms of Alzheimer’s disease [44], CBD was shown to be an effective treatment.

### 1.7 Spasticity

Many of the double-blinded, placebo-controlled studies for the effects of cannabinoids on spasticity used whole plant cannabis extracts or Sativex^®^ that is a 1:1 THC:CBD extract containing 2.5 to 120 mg THC and CBD/day. Visual Analogue Scale (VAS) scores for each patient's most troublesome symptom were significantly reduced [45].

### 1.8 Chronic Pain

In adults with chronic pain, patients treated with cannabis or cannabinoids are more likely to experience a clinically significant reduction in pain symptoms [24]. A recent review of specific cannabinoids and cannabinoid extracts on multiple pain types investigates both the preclinical and clinical data supporting cannabinoid pharmacotherapy for pain [46].

### 1.9 Cancer

There is tremendous interest in CBD as an anticancer agent. Aviello *et al*. showed that CBD had multiple chemopreventive effects in murine colorectal carcinoma cell lines by protecting DNA from oxidative damage, increasing endocannabinoid concentrations and reducing cell proliferation in a CB1-, TRPV1- and PPARγ-antagonists sensitive manner [47]. De Petrocellis *et al*. found that 1-10 µM CBD significantly inhibited human prostate carcinoma cell viability, inducing apoptosis and elevation of reactive oxygen species (ROS) [48]. Exciting new developments for enhancing CBD effects in inducing cell death and enhancing radiosensitivity of glioblastoma (GBM) cells were recently published [49]. GBM cells treated with CBD, γ-irradiation, and KU60019, an ATM kinase inhibitor, increased apoptosis and with strongly upregulated arrested cells, blockade of cell proliferation, and production of pro-inflammatory cytokines, improving CBD effectiveness.

### 1.10 Addiction Disorders

Recently, Solowij *et al*. described a 10-week study of daily 200 mg CBD in cannabis dependence to improve psychological symptoms and cognition [41]. CBD was well tolerated with no serious AE, promising therapeutic effects for improving psychological symptoms and cognition in regular cannabis users, and suggested that CBD may be a useful adjunct treatment for cannabis dependence. CBD improved subicular and CA1 subfields volumes in the brains of chronic cannabis users, suggesting a protective role of CBD against brain structural harms conferred by chronic cannabis use [40]. Moreover, CBD was shown to have low abuse liability [50, 51] and to be effective in decreasing cannabis addiction [52, 53].

### 1.11 The Current Context

In June 2018, the US FDA approved the marketing of Epidiolex^®^, a CBD-rich whole cannabis plant extract, for the treatment of seizures in patients over age two suffering from Lennox-Gastaut and Dravet syndromes, two drug-resistant forms of epilepsy with a higher early mortality rate [27]. The studies that led to FDA approval of Epidiolex^®^ for the treatment of severe forms of epilepsy, used CBD as an adjunct to clobazam, valproate, levetiracetam, and topiramate, resulting in seizures reduction with few AEs, compared to other drugs.

In January 2019, the World Health Organization (WHO) changed position after 60 years and proposed rescheduling of cannabis and cannabinoids for therapeutic purposes [54, 55]. Three months after FDA Epidiolex^®^ approval, the U.S. Drug Enforcement Administration (DEA) removed Epidiolex^®^ from the most restricted Schedule 1 (no approved medical use and high abuse liability) to Schedule V with low abuse potential [56].

In the wake of growing medical and public interest in medical cannabis and cannabinoids, we aimed to evaluate current knowledge of CBD’s AEs and toxicities by the relevant scientific literature from preclinical and clinical studies. Clinicians should be aware of CBD AEs and potential drug-drug interactions prior to recommending off-label CBD.

## 2 METHODS

A literature search, from inception to January 2019, was performed on PubMed, EMBASE, and CENTRAL (Cochrane Central Register of Controlled Trials) using the keywords cannabidiol, Epidiolex, adverse or side effects, adverse reactions or events, safety, complications, toxicity, and toxicology. Relevant articles were selected by the following criteria: articles acknowledging CBD AEs or toxicity, including studies focusing on the beneficial effects of the drug, and published in English. Several studies defining CBD’s beneficial effects were included to provide balance and aid the readers’ ability to weigh risk/benefit.

Further research manuscripts were retrieved through the reference lists of selected articles, and reports were found on international agencies or institutional websites including US FDA, WHO, US DEA, and US National Academies of Sciences, Engineering, and Medicine. All articles were screened independently by three co-authors to determine their relevance and included if selected by at least two co-authors.

## 3 RESULTS

CBD clearly has great potential as a new pharmacotherapy based on novel mechanisms of action for currently unmet clinical needs. However, CBD, like almost all medications, also produces AEs and toxicity. Two previous reviews focused on the therapeutic effects but also included AEs. In 2011, Bergamaschi *et al*. reviewed CBD AEs in animals and humans, concluding that CBD is generally safe, but further research is needed to investigate in-depth the observed *in vitro* and *in vivo* AEs [57]. In 2017, Iffland and Grotenhermen confirmed CBD’s safety profile, especially compared to other antiepileptics and antipsychotics [58]. These authors suggested that research should pursue AEs of chronic administration, hormonal effects, enzyme inhibition or induction, genotoxicity, drug transporters, and interactions with other drugs.

Currently, CBD is the focus of mass marketing campaigns and the subject of anecdotal reports claiming that CBD provides the answer for multiple illnesses from chronic pain to depression. Despite its Schedule I status in the US by the DEA, and lack of control by the FDA, CBD products are sold across the US and the internet. No medication should be prescribed or recommended until it is proven safe and effective for each indication under consideration. In addition, it is important to reflect whether the medication is safe for each individual based on his or her health, age, genetics, chronic illnesses, and other medications (due to the problem of drug-drug interactions). Now that Epidiolex^®^ is FDA-approved, off-label prescriptions will increase. The goal of this review is to inform clinicians, pharmacists, nurses, patients, public health authorities, and policymakers about CBD’s AEs, toxicities, and drug-drug interactions that should be evaluated prior to prescribing CBD.

Table **1** lists AEs identified in preclinical research, and Table **2**, AEs identified in clinical research. Both Tables **1** and **2** list AEs in chronological order.

### 3.1 Neurological Effects

#### 3.1.1 In Vitro Neurological Effects

*In vitro* CBD toxicity was identified in Sprague Dawley rats’ oligodendrocytes, the cells responsible for CNS white matter myelination [59]. Following incubation with 100 nM-10 μM CBD for 20-30 min, a concentration-dependent decrease in oligodendrocyte viability was observed. The mechanism appeared to be through increases in intracellular Ca^2+^. If there was no extracellular Ca^2+^, CBD-induced cell death was reduced at 1 µM by 50.4%±18%. Furthermore, the disruption of mitochondrial membrane potential (MMP), and ROS production were reduced. CB1, CB2, TRPV1, adenosine A2A, PPARγ, ryanodine, and inositol triphosphate (IP3) receptor antagonists did not prevent CBD-induced intracellular Ca^2+^ increase, suggesting that these receptors did not mediate these CBD actions. However, CBD toxicity at 1 µM was significantly impaired by caspase-inhibitors, poly(ADP-ribose) polymerase PARP-1 and calpain, suggesting caspase-dependent and -independent cell death pathway activation.

CBD's neuroprotective effect was investigated in human neuroblastoma SH-SY5Y cells during and after neuronal differentiation [60]. Terminally-differentiated cells incubated with 2.5 µM CBD were not protected against ROS produced by exposure to glycolaldehyde, methylglyoxal, 6-hydroxydopamine, and hydrogen peroxide. During SH-SY5Y cell differentiation, CBD did not induce changes in antioxidant potential, nor neurite density. CBD exposure during neuronal differentiation may sensitize immature cells to redox-active drug neurotoxicity.

#### 3.1.2 In Vivo Neurological Effects

In 1981, Rosenkrantz and Hayden investigated acute cannabinoid toxicity in rhesus monkeys following 150, 200, 225, 250, or 300 mg/kg intravenous (IV) CBD for 9 days [61]. The LD50 was 212 mg/kg CBD. Tremors were observed at all doses and CNS inhibition (depression, sedation, and prostration) was evident within 30 min.

There is considerable interest in the CBD treatment of schizophrenia. In a randomized, double-blind CBD versus amisulpride clinical trial (42 patients, CBD or amisulpride 200 mg/day increasing to 800 mg/day over 28 days), both treatments were shown to be safe and significantly associated with clinical improvement [38]. There were significantly fewer CBD AEs than for amisulpride, including fewer extrapyramidal symptoms (acute dyskinesias and dystonic reactions, tardive dyskinesia, Parkinsonism, akinesia, akathisia, and neuroleptic malignant syndrome). CBD treatment increased serum anandamide concentrations, possibly due to CBD inhibition of fatty acid amide hydrolase (FAAH).

McGuire *et al*. also found a low incidence (≥4%) of mild AEs including headache, with a frequency similar to placebo in schizophrenic patients [35]. However, a meta-analysis of CBD efficacy and safety in schizophrenia concluded that there was “moderate evidence” that CBD did not decrease symptoms and produced frequent AEs in patients, as measured by the PANSS, brief psychiatric rating scale (BPRS), and Stroop color-word test (SCWT) [62].

In 2017, Garberg *et al*. administered 50 mg/kg IV CBD to four piglets to evaluate drug safety and potential neuroprotective effects. CBD significantly reduced brain-derived neurotrophic factor (BDNF) expression and other signaling proteins in the hippocampus and frontal cortex with no effect in the striatum. It was concluded that CBD did not provide neuroprotection during early global hypoxia-ischemia [63]. However, in a study investigating possible treatments for neonatal hypoxic-ischemic encephalopathy, low 1 mg/kg IV CBD dose in combination with hypothermia, found neuroprotective effects and modulation of excitotoxicity and inflammation in newborn hypoxic-ischemic encephalopathy animal models [64].

The greatest success for CBD treatment is the reduction in seizures in children with refractive epilepsy. In a randomized, double-blind, placebo-controlled trial, CBD reduced atonic seizures in Lennox-Gastaut patients, who also received clobazam, valproate, lamotrigine, levetiracetam, or rufinamide [65]. Severe AEs occurred in 20 (23%) of 86 patients in the CBD group including sleep apnea. Twelve (14%) patients treated with CBD and one (1%) treated with placebo withdrew from the study.

Long-term CBD safety and efficacy were evaluated in children and adults with intractable epilepsies administered up to 10 antiepileptic drugs including clobazam, valproic acid, levetiracetam, lamotrigine, stiripentol, rufinamide, topiramate, and felbamate [66]. The starting oral CBD dose was 2‐10 mg/kg/day, escalating to 25‐50 mg/kg/day for a median 48-week duration. Twenty-four percent of 607 patients in the safety dataset (mean age 13 years) withdrew, primarily due to failed efficacy (n=89, 61%) and AEs (n=32, 22%). AEs were reported in 88% of all patients, severe AEs such as convulsions and status epilepticus were reported for 33% of patients.

In the Epidiolex^®^ FDA approval notification [27] and Epidiolex^®^ prescription information [19], CBD’s *in vivo* AEs in humans included, similar to other anti-epileptics, suicidal thoughts, suicide attempts, agitation, depression, aggression, and panic attacks.

### 3.2 Changes in Behavior

Most clinical CBD research focused on reduction in seizures in patients with Dravet’s or Lennox-Gastaut syndromes. The most common AEs were sedation, somnolence, fatigue, lethargy, and malaise. In an online survey of 117 parents who administered CBD cannabis preparations to their children with uncontrolled epilepsy, the median dose was 4.3 mg/kg/day for a median duration of 6.8 months [67]. AEs were reported in 59% of children, but there were no controls. Porter and Jacobson reported similar findings in another smaller online survey including 18 parents [68]. Drowsiness and fatigue reportedly affected 37% and 16% of children, respectively. In a retrospective study of 74 children 1–18 years old with seizures, the CBD dose ranged from 1 to 20 mg/kg/day for more than 3 months (average 6 months) [69]. AEs were reported in 47% of children. Status epilepticus was attributed to the disease, and drowsiness and fatigue could have been due to the other administered anti-epileptic drugs, making it difficult to assign AEs to the CBD treatment.

From 2016-2018, Devinsky *et al*. investigated CBD efficacy for the treatment of Dravet syndrome and Lennox-Gastaut syndrome and reported associated AEs. In a 2016 open-label clinical trial of 214 patients 1-30 years old with treatment-resistant epilepsy, patients received up to 25-50 mg/kg/day CBD for 12 weeks [70]. Of the 162 patients in the safety and tolerability analysis, 79% reported AEs, 25% somnolence, 11% convulsions, and more than 5% reported somnolence, fatigue, lethargy, convulsions, status epilepticus, changes in concentrations of concomitant antiepileptic drugs, gait disturbance, and sedation. Serious adverse events were reported in 30% patients, including one unexpected death regarded as unrelated to study drug. Twelve percent had severe adverse events possibly related to CBD use, the most common (6%) was status epilepticus. Ten percent receiving the highest dose had to lower the dose prior to the end of the trial and 4% stopped treatment, most likely due to AEs. The median reduction in monthly motor seizures was 36.5% (IQR 0–64.7).

In a 2017, randomized, double-blind, placebo-controlled CBD trial on Dravet’s syndrome, 120 children received 20 mg/kg/day oral CBD or placebo for 14 weeks, in conjunction with their standard treatment (1 to 5 antiepileptic drugs) [71]. AEs occurred more frequently in the CBD than the placebo group, with somnolence (36% vs 10%) being the most common AE. Another less common AE was fatigue.

Adverse reactions were reported in 199 children and young adults treated with 2-5 mg/kg/day CBD for uncontrolled seizures [72] and in 424 children and young adults treated with 0.5-50 mg/kg/day CBD for refractory epilepsy [73]. The most common AEs were drowsiness, somnolence, and fatigue.

In a 3-week 2018 treatment trial in 4 to 10-year-old children with Dravet’s syndrome receiving 5, 10, or 20 mg/kg/day CBD, there were more AEs following CBD than placebo [74]. Children were concomitantly taking clobazam, valproate, levetiracetam, topiramate, and stiripentol. The most frequent AEs were somnolence, sedation, ataxia, and abnormal behavior.

In a 2018 randomized double-blind trial investigating CBD effect on atonic seizures in 225 patients 2-55 years old with Lennox-Gastaut syndrome, patients received 10 and 20 mg/kg/day oral CBD for 28 days [75]. In conjunction with other antiepileptic drugs, seizure frequency was reduced compared to placebo. This most common AE was somnolence. Serious AEs included somnolence and lethargy. Somnolence occurred more frequently in those receiving 20 mg/kg/day CBD than 10 mg/kg/day. AEs were reported in 6 patients following 20 mg/kg/day CBD, one following the lower dose, and one receiving a placebo.

In a randomized, double-blind, placebo-controlled trial, CBD was efficacious in reducing atonic seizures in patients with Lennox-Gastaut syndrome, also taking clobazam, valproate, lamotrigine, levetiracetam, or rufinamide [65]. Treatment-related AEs, including somnolence, were mostly mild and occurred in 62% of 86 patients treated with 20 mg/kg/day CBD for 14 weeks. Severe AEs included sedation occurring in 23% of 86 patients receiving CBD; 14% patients treated with CBD and one (1%) treated with placebo withdrew from the study.

Long-term CBD safety and efficacy were evaluated in an ongoing expanded‐access program in children and adults with treatment‐resistant epilepsies receiving up to 10 antiepileptic drugs including clobazam, lamotrigine, topiramate, rufinamide, valproic acid, levetiracetam, stiripentol, and felbamate [66]. The starting oral CBD dose was 2‐10 mg/kg/day, escalating to 25‐50 mg/kg/day for a median 48-week duration. Twenty-four percent of 607 patients in the safety dataset (mean age 13 years) withdrew, mostly for lack of efficacy (n=89, 61%) and AEs (n=32, 22%). Eighty-eight percent experienced treatment‐emergent AEs, with the most common AE being somnolence (22%).

In a study on the efficacy of CBD in schizophrenia, there was a low incidence (≥4%) of mild AEs including somnolence and insomnia, with a frequency similar to that found in placebo [35].

Several preclinical and clinical studies documented CBD’s acute anxiolytic effects [76-78], although more recently ElBatsh *et al*. demonstrated that 10 mg/kg intraperitoneal (IP) CBD over 14 days produced an anxiogenic effect in rats [79].

CBD AEs in humans reported in the Epidiolex^®^ FDA approval notification [27] and the Epidiolex^®^ prescription information [19] included somnolence, sedation and lethargy, insomnia, sleep disorder and poor quality sleep, fatigue, malaise, and asthenia.

### 3.3 Hepatic Effects

Following 90 days of oral CBD (30-300 mg/kg/day), liver and kidney weights in rhesus monkeys were 13-56% greater than controls, without morphological changes in the organs [61].

In 214 patients 1-30 years old with treatment-resistant epilepsy receiving up to 25-50 mg/kg/day CBD for 12 weeks, 7% had slightly elevated liver function tests, but one had a significant increase in transaminases (considered hepatotoxic), leading to CBD withdrawal [70]. All patients with hepatic or platelet abnormalities were also taking valproate. In a 3-week treatment trial in 4 to 10-year-old children with Dravet’s syndrome receiving 5, 10, or 20 mg/kg/day CBD and concomitant anti-epileptic drugs, 6 patients taking CBD and valproate developed elevated transaminases, but not liver injury [74]. In a 2017, double-blind, randomized, placebo-controlled CBD trial on Dravet’s syndrome, 120 children and young adults received 20 mg/kg/day oral CBD or placebo for 14 weeks, along with standard treatment of 1 to 5 antiepileptic drugs [71]. AEs occurred more frequently in the CBD than placebo group including increases in liver-function tests. Patients with Lennox-Gastaut syndrome (n=225) receiving 10 and 20 mg/kg/day oral CBD for 28 days, reported serious AEs with elevated aspartate aminotransferase (AST), alanine aminotransferase (ALT), γ-glutamyltransferase (GGT) concentrations, and worsening chronic cholecystitis [75]. The most common AE was AST or ALT increases 3.2-12.2 times the upper limit of normal in 4 of 6 patients receiving 20-mg/kg/day CBD, one receiving 10-mg/kg/day CBD, and among patients concomitantly receiving valproate (79%, 9 in the 20 mg/kg/day group and 2 in the 10 mg/kg/day group). Overall, 9% receiving CBD had elevated liver AST concentrations and none in the placebo group. Severe AEs in Lennox-Gastaut patients receiving CBD treatment included increased ALT, AST, and GGT concentrations [65]. In children and adults with treatment‐resistant epilepsies receiving up to 25‐50 mg/kg/day for a median 48-week duration, AEs related to ALT/AST abnormalities (higher than three times the upper limit of normal) were reported for 10% of patients; 75% of these also received valproate [66].

CBD AEs in humans presented in the Epidiolex^®^ FDA approval notification [27] and Epidiolex^®^ prescription information [19] include transaminase elevation (especially with concomitant valproate). Epidiolex^®^ can also cause liver injury, usually mild, but more severe injury with related symptoms such as jaundice can occur although rarely.

### 3.4 Gastrointestinal Effects

In an online survey of 117 parents who administered a median CBD-enriched cannabis preparation of 4.3 mg/kg/day for a median duration of 6.8 months for treatment of their children's epilepsy, 59% reported AEs, primarily gastrointestinal disturbances; however, there was no control group [67]. In a retrospective study of 74 patients, age range 1-18 years, CBD dosage ranged from 1 to 20 mg/kg/day for more than 3 months (average 6 months), 47% AEs were reported, prominently [69]. Gastrointestinal disturbances could be due to other co-administered anti-epileptic drugs making it difficult to assign responsibility to CBD.

In 162 participants included in a clinical trial of 25-50 mg/kg/day CBD for 12 weeks for treatment-resistant epilepsy, serious AEs included diarrhea, weight loss, and gastrointestinal intolerance (n=1) [70]. In a CBD trial on Dravet’s syndrome, 120 children and young adults were randomly receiving 20 mg/kg/day oral CBD or placebo for 14 weeks, AEs included diarrhea (31% vs 10%), loss of appetite (28% *vs*. 5%), and much less commonly vomiting [71]. Diarrhea and weight and appetite loss were also reported in 199 children and young adults treated with 2-5 mg/kg/day CBD for uncontrolled seizures [72], and in 424 children and young adults treated with 0.5-50 mg/kg/day CBD for refractory epilepsy [73].

Similarly, in 4 to 10-year-old children with Dravet’s syndrome, CBD treatment with 5, 10, or 20 mg/kg/day reported appetite loss and vomiting as the most frequent AE [74]. Devinsky *et al*. reported AEs of decreased appetite, diarrhea, and vomiting, and a serious AE constipation in 2 to 55-year-old patients (n=225) with Lennox-Gastaut syndrome, receiving 10 and 20 mg/kg/day oral CBD for 28 days [75]. Decreased appetite and diarrhea occurred more frequently in the high-dose group than the low-dose group (10 mg/kg/day). CBD significantly reduced atonic seizures in patients with Lennox-Gastaut syndrome, with mostly mild AEs diarrhea, decreased appetite, and vomiting in 62% of 86 patients treated with 20 mg/kg/day CBD for 14 weeks [65]. Vomiting was among the severe AEs reported.

Following 25‐50 mg/kg/day for a median of 48 weeks in children and adults with treatment‐resistant epilepsies, 88% of all patients experienced treatment‐emergent AEs and 33% experienced severe AEs, including vomiting [66]. The most common AEs were diarrhea (29%), and decreased appetite 12%). In clinical studies of schizophrenia, mild AEs, diarrhea and nausea, occurred with a low incidence of ≥4%, with a frequency similar to placebo [35].

CBD AEs in humans listed in the Epidiolex^®^ FDA approval notification [27] and in Epidiolex^®^ prescription information [19] include decreased appetite, diarrhea, nausea, vomiting, and abdominal pain.

### 3.5 Drug-drug Interactions

CBD’s interaction with CYP enzymes can reduce or potentiate the effects of other drugs [19, 22, 80]. In 1974, Karniol *et al*. investigated effects of oral 0, 15, 30, and 60 mg CBD alone, 0 and 30 mg THC alone, and CBD and THC combinations to study potential drug-drug interactions in a double-blind trial in 40 healthy male volunteers [81]. THC alone disturbed time estimations, increased pulse rate, and induced strong psychological reactions, while up to 60 mg CBD alone produced no effects. Thirty to 60 mg CBD weakened or blocked time production impairment, psychological disturbances, and pulse rate acceleration produced by THC, when co-administered. CBD also decreased anxiety following THC, with subjects reporting more pleasurable effects.

In 1995, CBD effects on THC pharmacokinetics were investigated in mice receiving 120 mg/kg IV CBD 2 h before 12 mg/kg IV THC [82]. CBD inhibited hepatic microsomal THC metabolism reducing THC clearance. 7-OH-THC and 6α-OH-THC concentrations were increased in brain, with few changes in blood. CBD-induced changes in metabolite profile and brain pharmacokinetics might change pharmacological effects. Bergamaschi reviewed research on animal models in 2011 showing that CBD did not induce changes in food intake, catalepsy, or physiology in rats and mice [57]. Chronic low and high CBD doses inhibited hepatic drug metabolism producing drug-drug interactions *in vivo* in mice and rats following 10-120 mg/kg IP CBD [83-93]. However, more recently, when equal amounts of CBD and THC were co-administered, CBD did not modify THC blood concentrations in humans [94]. In addition, Karschner *et al*. found no changes in THC’s subjective and physiological effects when equivalent doses of THC alone or CBD and THC (Sativex^®^) were given *via* oromucosal spray [95].

In a clinical trial of treatment-resistant epilepsy, 162 participants included in the safety and tolerability analysis received 25-50 mg/kg/day CBD for 12 weeks and sustained changes in concentrations of concomitant antiepileptic drugs that may have led to status epilepticus [70]. Similarly, in patients with CBD-reduced atonic seizures, severe AEs increased concomitant antiepileptic concentrations in 23% of 86 patients in the CBD group; 14% of patients treated with CBD and one (1%) treated with placebo withdrew from the study [65].

THC, CBD, and THC and CBD effects *via* vaporization of 20 mg THC, CBD, or 1:1 THC:CBD, oral, and subcutaneous (SC) administration of 10 mg/kg THC or CBD, or 20 mg/kg 1:1 THC:CBD, or oral gavage were investigated in Wistar rats [96]. Although no statistical analyses were performed, SC CBD inhibited THC metabolism resulting in 4 times higher serum and brain THC concentrations when CBD and THC were simultaneously administered compared to THC alone. Serum and brain CBD concentrations were half the concentration when CBD and THC were co-administered compared to CBD alone. Oral CBD inhibited THC metabolism with 2 to 3 times higher serum and brain THC concentrations and two-fold lower serum and brain CBD concentrations when CBD and THC were administered together. CBD did not inhibit THC metabolism after pulmonary THC and CBD administration. SC cannabinoids administration (THC and CBD and THC and CBD alone) produced hypolocomotion. Oral THC and THC and CBD produced almost total immobility, but oral CBD produced mild hyperlocomotion. The apparent CBD inhibition of THC metabolism after oral and SC administration had little impact on THC-induced behavior.

In a CBD versus amisulpride trial, 42 patients significantly improved seizure control, with significantly higher serum anandamide concentrations, perhaps due to CBD inhibition of the enzyme FAAH [38].

Epidiolex^®^ and concomitant clobazam administration, produced a 3-fold increase in plasma concentrations of N-desmethylclobazam, the active metabolite of clobazam, increasing the risk of AEs such as excessive sedation [19, 97]. Epidiolex^®^ increases plasma concentrations of drugs metabolized by CYP2C19 such as diazepam or clobazam.

In 137 patients in the efficacy analysis receiving 25-50 mg/kg/day CBD for 12 weeks for treatment-resistant epilepsy, 11 withdrew due to AEs including allergy to sesame oil vehicle (n=1) [70]. In the Epidiolex^®^ FDA approval notification [27] and in Epidiolex^®^ prescription information [19], CBD AEs in humans included allergic reactions and rash.

### 3.6 Respiratory Effects

In 162 patients with treatment-resistant epilepsy receiving 25-50 mg/kg/day CBD for 12 weeks, a serious AE of pneumonia was reported [70]. In a trial investigating the effects of 10 and 20 mg/kg/day oral CBD for 28 days on atonic seizures in 2 to 55-year-old patients with Lennox-Gastaut syndrome (n=225), common AEs included upper respiratory tract infection [75]. In children and adults with treatment‐resistant epilepsies receiving up to 25‐50 mg/kg/day for a median 48-week duration, 33% experienced severe AEs including pneumonia [66]. The most common AEs were upper respiratory tract infection in 12%.

CBD AEs in humans in the Epidiolex^®^ FDA approval notification [27] and in the Epidiolex^®^ prescription information [19] included viral, fungal, and pneumonia infections.

### 3.7 Pyrexia

The most frequent AE was pyrexia in a 3-week treatment trial of 5, 10, or 20 mg/kg/day CBD in 4 to 10-year-old children with Dravet’s syndrome [74]. In the same manner, 2 to 55-year-old patients with Lennox-Gastaut syndrome (n=225), receiving 10 and 20 mg/kg/day oral CBD for 28 days for atonic seizures had pyrexia amongst common AEs [75]. Treatment-related AEs were mostly mild and occurred in 62% of 86 patients treated with 20 mg/kg/day CBD for 14 weeks, including pyrexia in patients with Lennox-Gastaut syndrome, with concomitant clobazam, valproate, lamotrigine, levetiracetam, or rufinamide [65].

### 3.8 Cardiovascular Effects

After rhesus monkeys received 150, 200, 225, 250, or 300 mg/kg IV CBD for 9 days, higher CBD doses elicited hypopnea, bradycardia, and cardiac failure [61]. In 162 patients with treatment-resistant epilepsy administered 25-50 mg/kg/day CBD for 12 weeks, serious AEs included diarrhea, weight loss, and gastrointestinal intolerance (n=1) [70]. Five (3%) patients experienced mild to moderate and one case of severe thrombocytopenia, resolving after stopping valproate. One patient also taking valproate developed hyperammonemia leading to stopping CBD intake. AEs were clearly related to dose and anti-epileptic drug intake. All patients receiving CBD and valproate had liver or blood abnormalities.

CBD was evaluated as a neuroprotectant after perinatal hypoxia-ischemia in piglets [63]. Piglets were randomized to 50 mg/kg IV CBD (n=13) or vehicle (n=9). CBD induced severe hypotension in two piglets; one suffered fatal cardiac arrest (50 mg/kg, IV). CBD (25 mg/kg, n=4) induced significant hypotension in one piglet, while 10 mg/kg (n=5) was well tolerated. A significant negative correlation between plasma CBD concentration and blood pressure during drug infusion was observed (p<0.005).

### 3.9 Reproductive Effects

#### 3.9.1 In Vitro Reproductive Effects

In 1982, the effects of 100-200 µM CBD reduced the basal accumulation of progesterone, testosterone, and estradiol-17*β* in preovulatory rat follicles by up to 60% [98]. Luteinizing hormone-stimulated increase in progesterone and testosterone was reduced by 75-88% following 50-200 µM CBD and estradiol-17*β* accumulation was inhibited by 40%.

Progesterone 17α-hydroxylase activity was significantly inhibited by 100-1000 µM CBD [99]. Testosterone 6*β* and 16〈-hydroxylase activity and androstenedione formation from testosterone in rat liver microsomes also were significantly reduced by CBD.

#### 3.9.2 In Vivo Reproductive Effects

Following oral 30, 100, and 300 mg/kg CBD for 90 days in rhesus monkeys, significant 57% decreases in testicular weights were observed after 200 mg/kg CBD that continued after the end of treatment [61]. Similarly, acute and subchronic 0.6, 0.8, and 1.2 mg/kg smoked CBD exposure in rats showed a severe dose-related seminiferous tubule degeneration with interference in sperm maturation [100]. In addition, testicular weight decreases correlated with a dose-related inhibition of spermatogenesis.

In 42 patients administered 200 mg/day gradually increased to 800 mg/day CBD or amisulpride over 28 days, there were significantly fewer CBD-related AEs compared to amisulpride, including lower prolactin release, and less sexual dysfunction [38].

Fertility also was affected by CBD. In sea urchins, *in vivo* fertilization was inhibited by 0.1-100 μM CBD due to a decreased acrosome reaction in sperm [101, 102].

When pregnant rats were administered 0, 75, 150, or 250 mg/kg/day oral CBD during organogenesis, developmental toxicity including increased embryofetal mortality at the highest dose was observed [103]. Oral 0, 50, 80, or 125 mg/kg/day CBD administration during organogenesis in pregnant rabbits decreased fetal body weights and increased fetal structural variations were shown following the highest dose [103]. Also, following 150 and 250 mg/kg/day oral CBD to pregnant and lactating rats, decreased growth, delayed sexual maturation, neurobehavioral changes with decreased activity, and AEs for male reproductive organ development and fertility in offspring were noted. No maternal toxicity was reported [103].

When Swiss mice received 30 mg/kg oral CBD or placebo in sunflower oil for 34 consecutive days, CBD decreased total circulating testosterone by 76% (still within normal ranges), significantly increased abnormalities in spermiation and meiotic stages [104]. CBD-treated mice had a 38% reduction in spermatozoa in the epididymis tail and more head abnormalities in the sperm and cytoplasmic droplets in the flagella medial region.

### 3.10 Cellular Effects

*In vitro* toxicity was observed in the production of cytokines in human eosinophil leukemia cells, peripheral blood mononuclear cells, human T-lymphotropic virus-1 (HTLV-1) positive B cells, and T cells following 1-10 μg/mL CBD [105]. CBD suppressed T-cell activities in splenocytes exposed to CBD *in vitro* or isolated from CBD-administered mice [106]. Exposure of splenocytes to CBD produced ROS, reduced cellular glutathione (GSH) content, and significantly stimulated caspase-8 activation. Pretreatment with a caspase-8 inhibitor significantly reduced, in a concentration-dependent manner, CBD-mediated apoptosis, but not ROS production, suggesting that CBD’s apoptotic effects in primary lymphocytes are associated with oxidative stress-dependent activation of caspase-8.

*In vitro* apoptosis was induced in mouse thymus and spleen cells exposed to 4-16 μM CBD [106, 107], and a pro-apoptotic effect was noted in lymphocytes following 10 mg/kg IP CBD [106].

ATP-binding cassette transporter (ABC) ABCG2 activity in mouse embryonic fibroblasts was reduced after *in vitro* exposure to 10-50 µM CBD [108].

Following exposure to 3-100 μM CBD, *in vitro* P-glycoprotein activity was reduced in human T lymphoblastoid leukemia cells [109]. In addition, ABCC1 transporter was inhibited in human ovarian carcinoma cells with a CBD IC50 of 128.3 μM [110]. CBD was also shown to interact with P-glycoprotein efflux transporters involved in multidrug resistance [111] and may also affect placental permeability and pharmacokinetics of other drugs. In humans, 600 mg of the antibiotic rifampicin, a CYP3A4 inducer involved in CBD metabolism, significantly reduced peak plasma CBD from 1.0 to 0.50 µg/L (-52%), while antifungal ketoconazole (400 mg), a CYP3A4 inhibitor, almost doubled peak plasma CBD from 0.7 to 1.3 µg/L (+89%) [112].

## 4 DISCUSSION

This is an exciting time for CBD research and medicine. Epidiolex^®^, containing 98% CBD, was approved by the FDA for the treatment of intractable epilepsy in patients with Dravet’s or Lennox-Gastaut syndromes, showing that a plant extract containing primarily CBD can provide the reproducibility needed for pharmacotherapies. There is active *in vitro* and preclinical research into the mechanisms of action of CBD in efforts to better understand its pharmacodynamics and pharmacokinetics and therapeutic potential. Clinical research is proceeding for multiple indications for CBD in well-designed, randomized, placebo-controlled clinical trials, by a variety of routes of administration. Pharmaceutical companies pursue synthetic CBD and plant extracts as CBD sources. CBD may provide a new approach as a stand-alone-drug and as an adjunct to other medications for unmet clinical needs.

Amongst all of these positive developments, unapproved CBD products are being sold across the US and in other countries without rigorous standardization of CBD potency, the content of other constituents, and with unproven claims of health effects. Now that Epidiolex^®^ is approved, it is likely that off-label prescriptions will increase. It is important that physicians and patients understand that CBD, like any other medication, is not appropriate for every individual and every disease and that it has side effects that are not negligible and must be considered prior to use.

The most important consideration is whether or not there is sufficient scientific data that CBD is efficacious in treating a patient’s disease or condition. The field is changing rapidly, but proof of efficacy is limited currently to CBD as an anti-epileptic. A second critical factor is dose, route, and frequency of administration. In many of the preclinical studies, much higher CBD concentrations were administered. For example, many of the cardiovascular, hepatocellular damage, inhibition of P450 systems, hormone changes, decreased fertility, alterations of *in vitro* cell viability, and reduced P-glycoprotein activities effects occurred at doses of >200 mg/kg/day [61], far above the current up to 50 mg/kg/day doses suggested in recent anti-epileptic clinical studies. However, in the clinical trial data to date, few cardiovascular and reproductive effects were reported. Other *in vivo* preclinical studies utilized lower doses similar to those used in humans, but the route of administration, IP or IV, provided higher bioavailability and hence, a greater chance of AEs and toxicity.

Drug interactions are an important issue to be carefully considered when prescribing CBD. CBD is often added to a regimen of other medications, especially other anti-epileptics and the potential for drug-drug interactions could lead to serious health consequences. *In vitro* and *in vivo* data suggest that CBD interacts with pharmaceuticals, specifically drugs metabolized by the liver. Drug-drug interactions with CYP1A2 substrates (theophylline, caffeine), CYP2B6 substrates (bupropion, efavirenz), UGT1A9 (diflunisal, propofol, fenofibrate), UGT2B7 (gemfibrozil, lamotrigine, morphine, lorazepam), and clinically significant interactions with CYP2C8 and CYP2C9 (phenytoin) substrates occur when co-administered with Epidiolex^®^ [19].

In humans receiving the drug for the treatment of epilepsies and psychiatric disorders, the most common AEs included tiredness, diarrhea, nausea, and hepatotoxicity. Overall, the incidence of these occurrences is low and, in comparison with other drugs employed for the treatment of these diseases, CBD has a better side effect profile.

The length of treatment is another important factor because data on AEs is much more limited following chronic CBD administration. Research is still needed on larger cohorts of CBD patients, and evaluation of CBD effects following long-term exposure on genotoxicity and cytotoxicity, hormones, and the immune system are needed.

Two of the common AEs after CBD administration are somnolence and sedation [19, 65, 70, 73]. These effects are dose-related and potentiated by co-administration of the anti-epileptic drugs including clobazam and valproate, and other CNS depressants (including alcohol). Patients should be advised that their ability to drive or operate machinery could be impaired while under CBD treatment.

From the patient’s point of view, it is particularly important to consider the proportions of THC and CBD in cannabis products when used for medical or recreational purposes, since self-medicating with cannabinoid products may expose patients to products with inaccurate labeling, containing impurities, underdosing or overdosing, insufficient supply, and risk of AEs and drug-drug interactions [41, 97]. This variability in CBD formulations (tablets, oromucosal spray, oral capsules, vaporized cannabis plant material, powder in oil, and CBD-THC products), and the wide CBD dose range (18-1500 mg) influence CBD efficacy and AEs [80].

## CONCLUSION

In conclusion, possible factors contributing to CBD AEs are CBD potency, route of administration (vaporized, transdermal, oral), concurrent licit and illicit drug use, and drug-drug interactions.

## Figures and Tables

**Table 1:** CBD adverse effects in preclinical studies.

**Species**	**CBD Dose**	**Route**	**Reported Adverse Effects (AEs)**	**Refs.**
**Acute AEs**				
Rats	0.6, 0.8, or 1.2 mg/kg	Inhaled	Organ weight elevation; Seminiferous tubule degeneration, interference in sperm maturation	Rosenkrantz and Hayden, 1979 [100]
Rhesus monkeys	150, 200, 225, 250, or 300 mg/kg/day (9 days)	Intravenous	Tremors, central nervous system inhibition, convulsions, bradycardia, hypopnea, cardiac failure at higher doses; Liver weight increase and testicular weights decrease, inhibition of spermatogenesis	Rosenkrantz and Hayden, 1981 [61]
Sea urchin eggs and sperms	0.1, 0.5, 1.0, or 10 µM	Incubation in CBD-enriched sea water	Dose-dependent decreased fertility of eggs & sperms & fertilization inhibition	Schuel *et al*., 1987 [101]
Rats	10 mg/kg	Intraperitoneal	Decrease of testosterone metabolism; Decrease of CYP aniline hydroxylation and p-nitroanisole demethylation, alteration of CYP contents	Narimatsu *et al*., 1990 [86]
Sea urchin sperms	0.1–100 µM	Incubation in CBD-enriched sea water	Dose and time-dependent acrosome reaction inhibition, motility not reduced	Schuel *et al*., 1991 [102]
Piglets	10, 25, or 50 mg/kg	Intravenous	Hypotension, cardiac arrest	Garberg *et al*., 2017 [63]
Rats	10 mg/kg + 10 mg/kg THC	Subcutaneous	THC metabolism inhibition with higher THC concentrations & lower CBD concentrations in serum and brain; Hypolocomotion: THC metabolism inhibition shows little to no impact on THC-induced behavior	Hložek *et al*., 2017 [96]
Rats	10 mg/kg + 10 mg/kg THC	Oral	THC metabolism inhibition with higher THC concentrations & lower CBD concentrations in serum and brain; Almost total immobility (10 mg/kg CBD alone caused mild hyperlocomotion): THC metabolism inhibition shows little to no impact on THC-induced behavior	Hložek *et al*., 2017 [96]
Rats	10 mg + 10 mg THC (5 min vaporization)	Inhaled	No THC metabolism inhibition	Hložek *et al*., 2017 [96]
**Chronic AEs**				
Rhesus monkeys	30, 100, or 300 mg/kg/day (90 days)	Oral	Liver, heart, kidney, and thyroid weight increase; Decrease in testicular size, spermatogenesis inhibition	Rosenkrantz and Hayden, 1981 [61]
Rats	10 mg/kg (14 days)	Intraperitoneal	Anxiogenic-like effect, decreased brain-derived neurotrophic factor (BDNF) expression & related signaling proteins in the hippocampus and frontal cortex; Protein expression decrease in animals with enhanced protein expression following chronic antidepressant/anxiolytic drug treatment	ElBatsh *et al*., 2012 [79]
Mice	30 mg/kg (15 days)	Intraperitoneal	Decreased cell proliferation and neurogenesis in the hippocampus and in subgranular zone	Schiavon *et al*., 2016 [36]
Rats (pregnant)	75, 150, or 250 mg/kg/day (during organogenesis)	Oral	Developmental toxicity, increased embryofetal mortality	Center for Drug Evaluation and Research, 2018 [103]
Rats (pregnant)	75, 150, or 250 mg/kg/day (during pregnancy and lactation)	Oral	Decreased growth, delayed sexual maturation, neurobehavioral changes, alterations of male reproductive organ development & fertility in offspring	Center for Drug Evaluation and Research, 2018 [103]
Rabbits (pregnant)	50, 80, or 125 mg/kg/day (during organogenesis)	Oral	Decreased fetal body weight, increased fetal structural variations	Center for Drug Evaluation and Research, 2018 [103]
Mice	15 or 30 mg/kg (34 days)	Oral	Decreased circulating testosterone, increased frequency of mitotic stages I-VI, decrease in spermiation stages VII-VIII & meiotic stage XII, decrease in number of Sertoli cells at meiotic stage (XII), decrease in number of spermatozoa in the epididymis tail, head abnormalities in sperm, cytoplasmic droplets in the flagella medial region	Carvalho *et al*., 2018 [104]

**Table 2:** CBD adverse effects in clinical studies.

**Study ** **Characteristic**	**Patients' ** **Characteristic**	**Oral CBD Dose**	**Simultaneous Drug ** **Administration**	**Reported Adverse Effects (AEs)**	**Refs.**
**Neurological studies**					
Parental report: online survey	Age 2-16; 18 patients with Dravet syndrome, Lennox-Gastaut syndrome, Doose syndrome, or idiopathic epilepsy	0.5–28.6 mg/kg/day (2 weeks–12 months)	Not reported	Moderate (defined as: sufficiently discomforting so as to limit or interfere with daily activities and may require interventional treatment): drowsiness (37%), fatigue (16%)	Porter and Jacobson, 2013 [68]
Parental report: online survey	Age 3-10; 117 patients with Dravet syndrome, Lennox-Gastaut syndrome, or infantile spasms	Median of 4.3 mg/kg/day (6.8 months)	Clobazam, other not-specified antiepileptics	AEs in 59% patients; Moderate: increased appetite, weight gain, drowsiness	Hussain *et al*., 2015 [67]
Open-label study, expanded-access trial in 11 independent epilepsy centers	Age 1-30; Patients with treatment-resistant epilepsy; 162 patients in safety analysis group (33 with Dravet syndrome, 31 with Lennox-Gastaut syndrome)	2–5 mg/kg/day increased until intolerance or to a maximum of 25–50 mg/kg/day (12 weeks)	Clobazam, valproate	AEs in 79% safety group patients (128/162); Moderate: somnolence, fatigue, lethargy, sedation, decreased or changes in appetite, diarrhea, transaminases increase, changes of antiepileptics serum concentration; Severe: status epilepticus, convulsions, diarrhea, weight loss, thrombocytopenia, hyperammonaemia, hepatotoxicity	Devinsky *et al*., 2016 [70]
Retrospective study with no control group	Age 1-18; 74 patients with treatment-resistant epilepsy	1–20 mg/kg/day; 81% patients (60/74) with < 10 mg/kg, 19% (14/74) with >10 mg/kg (> 3 months, average 6 months)	Not reported	AEs reported in 47% patients (34/74); Moderate: seizure aggravation (5 patients stopped CBD treatment due to seizure aggravation), somnolence, fatigue, gastrointestinal disturbances, irritability	Tzadok *et al*., 2016 [69]
Double-blind, randomized, placebo-controlled trial	Age 2-18; 120 patients with Dravet syndrome	20 mg/kg/day (14 weeks)	Median of 3 antiepileptics (*e.g*., clobazam, valproate)	AEs in 93% patients; Moderate: diarrhea, loss of appetite, lethargy, fatigue, pyrexia, convulsion, elevated aminotransferase levels, somnolence; Severe (10 patients): elevated levels of liver aminotransferase enzymes (n=3), status epilepticus (n=3)	Devinsky *et al*., 2017 [71]
Double-blind, randomized, placebo-controlled trial	Age 4-10; 34 patients with Dravet syndrome	5, 10, or 20 mg/kg/day (4-week baseline, 3-week treatment, 10-day taper, and 4-week follow-up)	Clobazam, valproate, levetiracetam, topiramate, stiripentol	Treatment-emergent AEs (TEAEs) reported in 80% patients with 5 mg/kg (8/10), 63% patients with 10 mg/kg (6/8), 78% patients with 20 mg/kg (7/9), 86% patients with placebo (6/7); Moderate: pyrexia, sedation, somnolence, appetite loss, vomiting, ataxia, abnormal behavior, rash; Severe: pyrexia, maculopapular rash, elevated transaminases	Devinsky *et al*., 2018 [74]
Double-blind, randomized, placebo-controlled trial	Age 2-55; 225 patients with Lennox-Gastaut syndrome	10 or 20/mg/kg/day (28 days)	Not-specified antiepileptics	AEs in 84% patients with 10 mg/kg (56/67), in 94% patients with 20 mg/kg (77/82); Moderate: somnolence, decreased appetite, diarrhea, upper respiratory tract infection, pyrexia, vomiting; Severe: elevated aspartate aminotransferase (AST) concentration, elevated alanine aminotransferase (ALT) concentration, elevated γ-glutamyltransferase concentration, somnolence, increased seizures during weaning, nonconvulsive status epilepticus, lethargy, constipation, worsening chronic cholecystitis	Devinsky *et al*., 2018 [75]
**Neurological studies**					
Double-blind, randomized, placebo-controlled trial	Age 2-55; 171 patients with Lennox-Gastaut syndrome	20/mg/kg/day (14 weeks)	Clobazam, valproate, lamotrigine, levetiracetam, rufinamide	AEs in 62% patients (53/86); Moderate: diarrhea, somnolence, pyrexia, decreased appetite, vomiting; Severe: increased ALT concentration, increased AST concentrations, increased γ-glutamyltransferase concentrations	Thiele *et al*., 2018 [65]
Ongoing expanded‐access program (EAP)	Age 0.4-62 (average 13); 607 patients with treatment-resistant epilepsy	2-10 mg/kg/day increased to a maximum of 25-50 mg/kg/day; median duration 48 weeks	Up to 10, including clobazam, lamotrigine, topiramate, rufinamide, valproate, levetiracetam, stiripentol, felbamate	AEs in 88% patients; Moderate: diarrhea, somnolence, convulsions; Severe (33%): convulsions, status epilepticus, liver abnormalities (10%)	Szaflarski *et al*., 2018 [66]
**Psychiatric studies and psychiatric AEs**					
Double-blind, randomized CBD versus amisulpride trial	Age 18-50; 42 patients with acute paranoid schizophrenia or schizophreniform psychosis	200 mg/day increased to a maximum of 800 mg/day (28 days)	Lorazepam	Fewer motor disturbances, weight gain, and sexual dysfunction than amisulpride	Leweke *et al*., 2012 [38]
Meta-analysis of studies & reviews on CBD efficacy & safety in schizophrenia	57 patients with schizophrenia	300–600 mg	Not reported	Does not decrease anxiety; Frequent AEs (not reported)	Guinguis *et al*., 2017 [62]
Double-blind, randomized, placebo-controlled trial	Age 18-50; 32 patients with persecutory ideation and anxiety	600 mg	Not reported	AEs in 31% patients (5/16); Tiredness/sedation (n=5), lightheaded/dizziness (n=2), nausea (n=2), abdominal discomfort (n=1), increased appetite/hunger (n=2); A strong trend toward increased anxiety was documented; no effect on persecution ideation	Hundal *et al*., 2018 [39]
Double-blind, randomized, placebo-controlled trial	Age 18-65; 88 patients with no treatment-resistant schizophrenia or related psychotic disorder	1,000 mg/day (43±3 days)	Not-specified antipsychotics	AEs in 35% patients (15/43) (similar as placebo); Moderate: diarrhea (n=4; placebo, n=2), nausea (n=3), headache (n=2, placebo, n=2)	Mc Guire *et al*., 2018 [35]
